# Entropic Effects in Polymer Nanocomposites

**DOI:** 10.3390/e21020186

**Published:** 2019-02-15

**Authors:** Xiaobin Dai, Cuiling Hou, Ziyang Xu, Ye Yang, Guolong Zhu, Pengyu Chen, Zihan Huang, Li-Tang Yan

**Affiliations:** State Key Laboratory of Chemical Engineering, Department of Chemical Engineering, Tsinghua University, Beijing 100084, China

**Keywords:** entropy, nanocomposites, thermodynamics, nanostructure, morphology

## Abstract

Polymer nanocomposite materials, consisting of a polymer matrix embedded with nanoscale fillers or additives that reinforce the inherent properties of the matrix polymer, play a key role in many industrial applications. Understanding of the relation between thermodynamic interactions and macroscopic morphologies of the composites allow for the optimization of design and mechanical processing. This review article summarizes the recent advancement in various aspects of entropic effects in polymer nanocomposites, and highlights molecular methods used to perform numerical simulations, morphologies and phase behaviors of polymer matrices and fillers, and characteristic parameters that significantly correlate with entropic interactions in polymer nanocomposites. Experimental findings and insight obtained from theories and simulations are combined to understand how the entropic effects are turned into effective interparticle interactions that can be harnessed for tailoring nanostructures of polymer nanocomposites.

## 1. Introduction

Entropy (*S*), the measure of possible states that a system can take, has drawn intense research interest in polymer nanocomposites (PNCs), owing to their rich fundamental behaviors associated with self-assembly [1,2,3,4,5,6], stimulus-response [7,8,9], and so on. It is clear that entropy of an equilibrated, isolated ideal gas has been estimated as the Boltzmann’s equation, which is calculated as

*S*_eq_ = *k*_B_*lnW*, (1)

where *W* is the number of microscopic states and *k*_B_ refers to Boltzmann constant. The concept associated with randomness and probability distribution (Shannon definition) gives a well-defined meaning in thermodynamics. According to Helmholtz free energy function,

*ΔF = ΔU−TΔS* (2)

a physical system spontaneously minimizes its free energy, so it is important to identify a variety of mechanisms involving changes in enthalpy and entropy. 

One of the most characteristic features of soft matter is that it is organized on mesoscopic length scales, where the basic structural units are organized on a mesoscopic scale into entities that are much larger than an atom but still much smaller than the overall size of the material. However, the relatively weak interactions between them cause the characteristic softness of these materials, which lead to sensitivity toward external stimuli, associated with a slow response with long relaxation times [10]. In contrast with enthalpic penalty for non-bound interactions, such as van der Waals force, hydrogen bonding, ionic complexion, and π–π interactions, entropic penalty associated with large amounts microscope states or configurations is around a few *k*_B_*T*, which is essential for considering the entropic contribution to structural organization of various soft matter systems [2].

**Figure 1:**
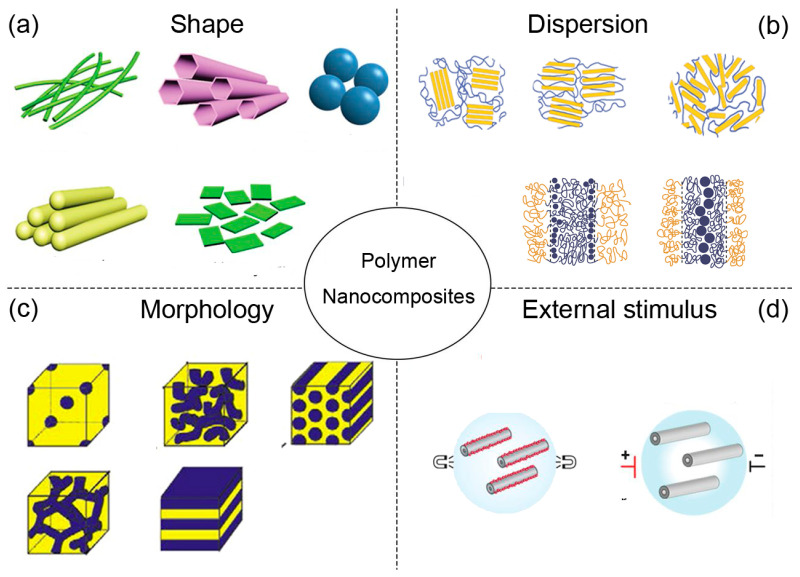
Research topics in polymer nanocomposites, where (**a**) shape, (**b**) dispersion, (**c**) morphology, and (**d**) external stimulus.

Over the last decades, polymer nanocomposites have demonstrated a tremendous increase in research, as they provide substantial property enhancements even at very low filler loadings, showing great prospects in mechanical [11,12,13,14], optical [9], and electrical [15,16,17,18] materials. Simultaneously, amazing advances have been taken in synthesis of various shapes of nanoparticles and order nanostructures that have revolutionized developments of advanced nanomaterials with unprecedented properties [19]. One of the most common strategies that is able to form an ordered structure precisely is self-assembly, which is a type of bottom-up approach that involves individual components following independent instructions to collectively form hierarchical structures, overcoming the limitations that a top-down approach may have, such as inherent size, parallelization, and 2-dimensional (2D) processing [20,21,22]. However, notable property enhancements in nanocomposites are difficult to achieve, due to the morphology of the polymer matrix and dispersion of nanoparticle controlled by both thermodynamics and kinetics. As a consequence, the increased interest in entropy is driven by the development of new polymer nanocomposite materials and guided by the following items: (a) shape, (b) dispersion and location, (c) morphology, and (d) external condition, as illustrated in Figure 1.

**Figure 2:**
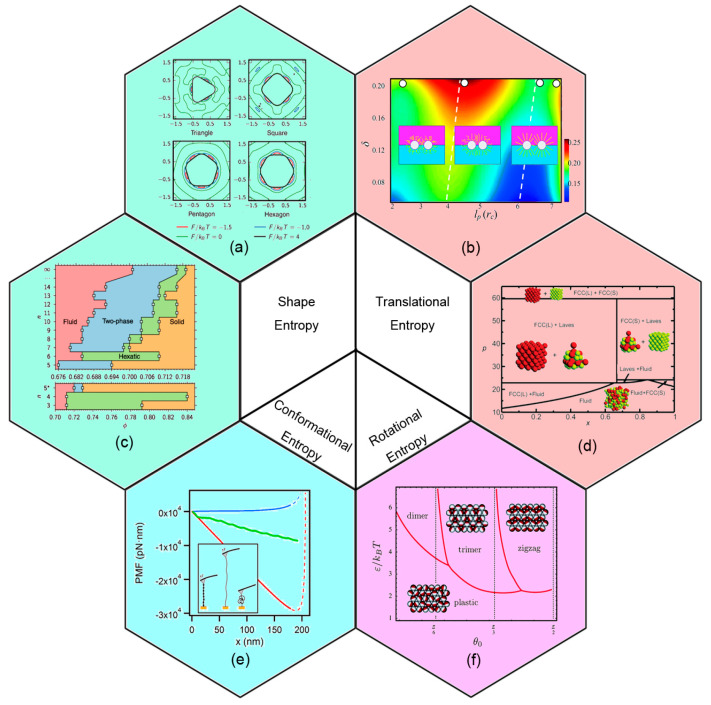
Typical entropy types used in self-assembly. (**a**) A contour plot of potential of mean force and torque plots for polygons at the highest density point of isotropic fluid. (**b**) Mixing parameter as a function of persistence length of tethers *l*_p_, and the compression ratio caused by mixing entropy. Reproduced with permission from ref. [31] Copyright 2013, The Royal Society of Chemistry. (**c**) Phase diagram of hard polygon melting behavior, which is controlled by shape entropy. (**d**) Phase diagram for a binary hard-sphere mixture with size ratio q = 0.82 in the reduced pressure *p* and the number fraction of small spheres *x*. Reproduced with permission from ref. [25] Copyright 2015, The Springer Nature. (**e**) Conformation and elasticity of modular proteins at different external force condition. The free energy landscape (FEL) of unfolding (green line), stretching (red line), and collapse-refolding (blue line) conformation of polyubiquitin was measured by AFM. Reproduced with permission from ref. [33] Copyright 2017, The American Chemical Society. (**f**) Patchy particles rotates to assemble. Phase diagram in terms of the patch size *q*_0_ and reduced attraction strength *ε*/*k*_B_*T*, at a reduced pressure *p* =50. Reproduced with permission ref. [32] Copyright 2015, The Royal Society of Chemistry. (a) and (c) are reproduced with permission ref. [34] Copyright 2017, The American Physical Society.

Though the most popular metaphoric description of entropy is disorder, entropic ordering transitions have indeed been identified in many soft matter systems, including colloids [23,24,25], glasses [26,27,28], and particularly polymer nanocomposite systems [29,30,31]. Furthermore, different manifestations of entropy are classified according to particular degrees of freedom of molecules [6], as shown in Figure 2, such as translational entropy [9,31], rotational entropy [22,32], conformational entropy [29,33], and shape entropy [34,35,36,37], taking advantage of the existence of such an entropy-driven ordering transition. A well-known example, predicted by Onsager theory [38], is the phase transition from the isotropic fluid to the nematic liquid crystal phase for hard rods with small aspect ratio, in which the loss of orientational entropy is compensated by a much higher gain of translational entropy from excluded volume between pairs of rods [6,39]. The physical processes where entropy increase results in a more ordered thermodynamic system may serve as food for emerging topics of soft matter researches.

Understanding the complicated entropic effects has been an ongoing challenge for many decades. Based on large amounts of experimental evidences [40,41] and multistate simulation results [29,42], a series of theories [30,43,44,45,46,47] bring about some excess practical explanations that help to understand the entropic order transition and design more fascinating materials. For example, entropic depletion force was first introduced by Asakura and Oosawa [44,45], which is described as excess translational entropy gained from the overlap of an excluded volume of particles, successfully explaining aggregation of large hard spheres dissolved in solution. A recent review [48] has summarized many types of entropy that tailored the structure formation and interface properties of materials. However, the entropic and overall thermodynamic landscape can be further complicated by inducing additional perturbations to the systems. In this review, we thereby discuss the subtle entropic effects in polymer nanocomposites, combining experimental findings with insight obtained from theories and simulations.

## 2. Methods and Models of Molecular Simulations

In order to promote understanding of thermodynamics properties that can play a significant role in nanocomposites, theoretical and numerical simulations based on the fundamental physics with multiple length and time scales are becoming increasingly vital, supporting experiments with significant and foreseeable information [49]. Particle-based methods such as molecular dynamics (MD) and Monte Carlo (MC) are in the most common use to obtain a sufficient description of the representative items mentioned in *Introduction*.

### 2.1. Molecular Dynamics

Molecular dynamics (MD) is an effective method to study the microscopic properties of a material, based on Newton’s second law or the equation of motion,

**F**=m**a**, (3)

where **F** is the force vector exerted on the particle, *m* is the mass, and **a** is the acceleration vectors. In terms of the force field derived from the potential of the interactions among particles, it is possible to yield a trajectory that describes the positions, velocities, and accelerations of the particles as they vary with time [50]. Thus, the particle-based MD simulation has the ability to provide real molecular structural and dynamics details. However, the computational cost of MD simulations using the atomistic model is too large to afford, and thus coarse-grained models using a interacting particle to represent a few monomers constituting the polymeric chains have been widely adopted in large scale computation [51].

To conduct the molecular dynamics (MD) simulations in the canonical ensemble (*NVT*), a thermostat is indispensable to modulate the temperature in some fashion, such as Nose-Hoover thermostat, Langevin thermostat, and dissipative particle dynamics (DPD) thermostat [51]. 

Langevin thermostat can mimic the effects of the implicit solvent, assuming that particles being simulated are surrounded by a lot of much smaller fictional solvent particles, providing the friction force **F**_*i*_^F^ which is taken as Stokes equation,

(4) $$F_{i}^{F}=-\xi \frac{dr_{i}}{dt}=-3\pi d\eta \frac{dr_{i}}{dt},$$

where **r**_i_ is the position vector of particle *i*, *ξ* is the friction coefficient, *d* is the particle diameter, and *η* is the viscosity. Random force **F**_*i*_^R^ is taken as fluctuation dissipation theorem,

(5) $$\langle F_{i}^{R}(t)F_{i}^{R}(t^{\prime })\rangle =6k_{B}Tm\zeta \delta_{ij}\delta (t-t^{\prime }),$$

where *δ* is Kronecker delta, and the temporal evolution of particle m**a**_i_=**F**_*i*_^F^ + **F**_*i*_^R^ + **F**_*i*_^C^, where the conservative force **F**_*i*_^C^ is derived by the potential of the interactions among particles. It should be noted that the momentum is not conserved in the formulation of fluctuation dissipation theorem. As a result, Langevin thermostat cannot reproduce correct hydrodynamics and is limited to the prediction of diffusion properties, for instance a shear flow [52].

DPD thermostat has been introduced to overcome the entanglement effect of polymer that slows down the dynamics, which is available for vast MD simulations [53]. For the DPD thermostat, the pairwise random and dissipative forces to the force term of the Hamiltonian equations of motion which is given by,

(6) $$m_{i}a=F_{i}^{C}+F_{i}^{D}+F_{i}^{R},$$

The conservative force **F**_*i*_^C^ is a sum of the harmonic springs $F_{i}^{C_{b}}$ and the soft repulsion $F_{i}^{C_{\mathrm{nb}}}$:

(7) $$F_{i}^{C_{\mathrm{nb}}}=\{\begin{array}{c} \alpha_{\mathrm{ij}}\omega (r_{\mathrm{ij}})e_{ij}r_{\mathrm{ij}}\;<\;r_{c}\;\\ 0r_{\mathrm{ij}}\;\geq \;r_{c} \end{array}F_{i}^{C_{b}}=-K\left(r_{ij}-r_{0}\right)e_{ij},$$

where **e**_ij_= **r**_ij_ /|**r**_ij_*|*, *α*_ij_ is the maximum repulsion between particles *i* and *j*, *K* is the bond constant, *r*_0_ is the equilibrium bond length, *r*_c_ is the cut-off length, and the weight function *ω*(*r*_ij_) is chosen as

(8) $$\omega (r_{\mathrm{ij}})=\{\begin{array}{cc} 1-r_{ij}{/r}_{c} & r_{\mathrm{ij}}\;<\;r_{c}\\ 0 & r_{\mathrm{ij}}\;\geq \;r_{c} \end{array},$$

The dissipative and random forces **F**_*i*_^D^, **F**_*i*_^F^ are expressed respectively by

(9) $$F_{i}^{D}=-\xi \omega \left(r_{ij}\right)\left(v_{i}\cdot e_{ij}\right)e_{ij},$$

(10) $$\langle F_{i}^{R}(t)F_{i}^{R}(t^{\prime })\rangle =6k_{B}Tm\zeta \delta_{ij}\delta (t-t^{\prime }).$$

The fact that it neglects the entanglements and speeds up the simulations is correct, but it is also a big shortcoming of DPD in polymer simulations. In fact, many groups have worked hard to resolve this issue in the last decade [54,55]. To get a better understanding of molecular dynamics, a recent review has provided systematical comparison of those methods in terms of accuracy, efficiency, and stability both in equilibrium and non-equilibrium settings [52].

### 2.2. Monte Carlo

In MC simulations, the position of particles repeats random sampling at every step [56]. A frequently used importance sampling algorithm in equilibrium state is the Metropolis algorithm, originally derived for the specific case of the Boltzmann distribution [57]. When the change of energy approximated by the potential is lower than an arbitrarily chosen threshold, the positional vector updates, so the probability of accepting a move is given by,

(11) $$P_{accept,n}=\min\{1,\exp(-\beta [U(r_{n}^{N})-U(r_{n-1}^{N})])\},$$

where *U* is the potential energy, **r***^N^* is a collection of the positions of all *N* particles, and *β* = 1*/k*_B_*T* represents reciprocal energy unit. The probability density of finding the system is given by the expression [49],

(12) $$\rho (r^{N})=\frac{\exp[-\beta U(r^{N})]}{\int dr^{N}\exp[-\beta U(r^{N})]},$$

and the average value of some property <A> can be evaluated by the expression,

(13) $$\langle A\rangle =\frac{\int dr^{N}\exp[-\beta U(r^{N})]A(r^{N})}{\int dr^{N}\exp[-\beta U(r^{N})]}.$$

Compared with molecular dynamics method, MC simulations are free from the restrictions of solving Newton’s equations of motion, which allows for cleverness in the proposal of moves that generate trial configurations within the statistical mechanics ensemble of choice. MC is an accurate and reliable approach to evaluate thermodynamics properties at equilibrium, including an estimation of enthalpy and entropy, when converged to the limiting condition. However, there remain important practical problems which limit the applicability of the Monte Carlo approach. For non-equilibrium systems [58], such as statistical mechanical hopping models with driven dynamics [59], sheared soft matter systems [60] and chemical models of gene regulatory circuits [61], the relative probabilities of microstates *ρ*(**r***^N^*) are quite different from those of equilibrium states [62], and it often falls into the local minimum when the method is applied to research the system with a great number of metastable structures. Thus, a series of efficient sampling methods for non-equilibrium steady states [63,64] are developed which are not based on Equations (12) and (13), but instead take alternative approaches. Another problem is no dynamical information can be gathered from a traditional Monte Carlo simulation. This has motivated interest in a hybrid Monte Carlo (HMC) model for structure and property prediction [65,66], which adopts physical system dynamics rather than a probability distribution to predict future microstates.

## 3. Entropy-Induced Transition Due to Nanoparticles

The systematic studies of polymeric nanocomposite systems by experiments and computational simulations have produced overwhelming evidence that size, topology, and volume fraction of nanoparticles have a significant effect on structural formation of those materials, accounting for entropic contributions [51]. In the following examples, we note the trends that are described, such as dispersion and segregation of nanoparticles, the alignment and arrangement of grafted polymers to the surfaces, and the morphology of microscopic phases, which results from a drastic increase or decrease in entropy.

### 3.1. Particle Size

**Figure 3:**
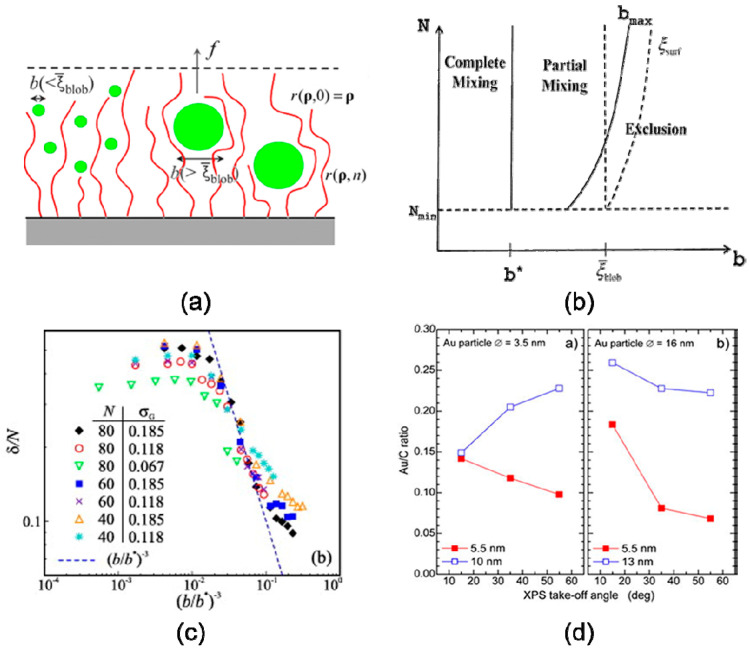
Polymer-soluble nanoparticles disperse in the film of substrate-grafted polymers. (**a**) Schematic of polymer film containing nanoparticles in contact with air. (**b**) Predicted phase diagram describing polymer brush–nanoparticle mixtures: complete mixing *b* < *b*^∗^, partial mixing *b*^∗^ < *b* < *b*_max_, exclusion *b* > *b*_max_. The typical blob size and surface blob size are *ξ*_blob_ and *ξ*_surf_, respectively. Reproduced with permission ref. [30] Copyright 2009, The American Physical Society. (**c**) Variation of the penetration length *d*/*N* with dimensionless inclusion size (*b*/*b*^∗^) for brushes of different height *L* and grafting density *s*, given in the legend. The (*b*/*b*^∗^)^-3^ is indicated by a dashed line. Reprinted from ref. [43], with permission from Elsevier. (**d**) Gold-to-carbon (Au*/*C) elemental ratio as a function of the XPS take-off angle for the two nanoparticle sizes in polymer brushes with two different thicknesses. Reprinted from ref. [40], with permission from IOP Publishing. Copyright 2003, IOP Publishing.

Nanoparticle size can have a significant effect on their localization within the phase domains. Following the works of Alexander [46] and de Gennes [47] regarding the use of a scaling approach to study a planar polymer brushes system, Kim and O’Shaughnessy [30,67] presented a size-dependent theory of dry polymer brush containing nanoparticles, shown in Figure 3a, b. The theory based on SCFT suggested that for the equilibrium particle penetration depth *δ* and the mean density of nanoparticle ${\bar{\phi }}_{\mathrm{nano}}$ scale as predicted,

(14) $${\bar{\phi }}_{\mathrm{nano}}(b)\propto {\left(b^{*}/b\right)}^{3},$$

(15) $$\delta (b)\propto {\left(b^{*}/b\right)}^{3}$$

indicating that small particles disperse freely within the polymer brush while large nanoparticles tend to aggregate at the brush-air interface. Yaneva et al. [43] used DPD to perform numerical simulations of the drying brushes by varying the length of the chains *N*, the grafting density where the resulted scale laws are merely in agreement with theoretical prediction, shown in Figure 3c. An increase in particle size leads to an increase in steric repulsion around nanoparticles [2] which undergo a decrease in conformational entropy when swelled in the polymeric domain, inducing an excluded entropic force. However, if nanoparticles are highly compatible with functional groups of grafted chains, the enthalpic interaction can be strong enough to overcome the entropic-induced repulsion by the polymer brush and the nanoparticles are uniformly “dissolved” in the brush. These predictions were demonstrated by experiments [40] of poly(acryl amide) (PAAm) grafted on flat silica surfaces. Figure 3d demonstrates the gold-to-carbon (Au/C) ratio as a function of XPS take-off angle (associated with submerging depths of nanoparticles), showing that gold nanoparticles are located closer to the PAAm brush/air interface for thin brushes, but small particles were able to penetrate the thick brushes. 

**Figure 4:**
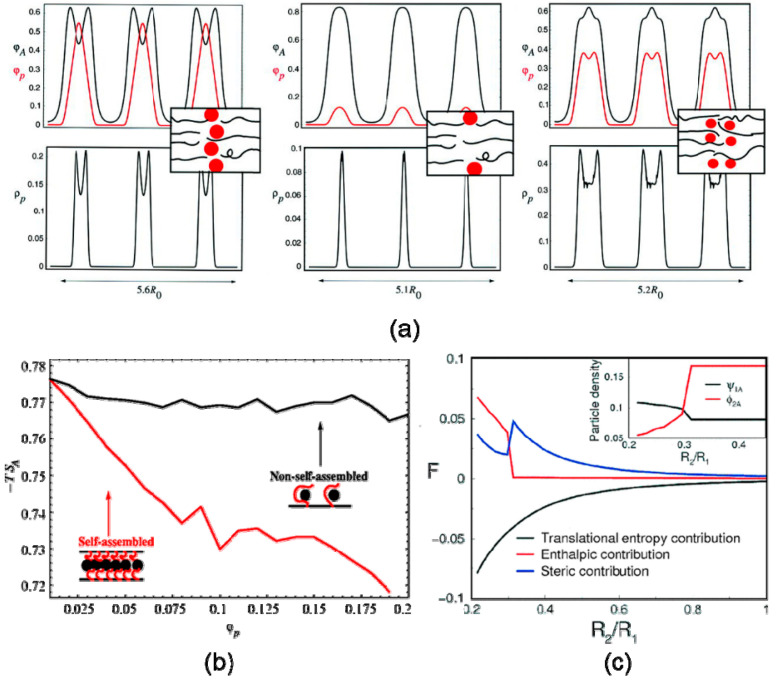
SCF/DFT calculations for preferential nanoparticles dispersed in the symmetric diblock copolymer matrix. (**a**) Spatial distribution of nanoparticles in different sizes and concentrations: large and concentrated (left), large and dilute (middle), small and concentrated (right). (**b**) Entropy contribution as a function of density profile of nanoparticle, where large particles (red line) self-assemble while small particles (black line) don’t, from ref. [28]. Reprinted with permission from AAAS. (**c**) Enthalpy, conformational (steric) entropy, and translational entropy contribution to free energy as a function of reduced particle size R_2_/R_1_. Reprinted with permission from ref. [54] Copyright 2002 by the American Physical Society.

**Figure 5:**
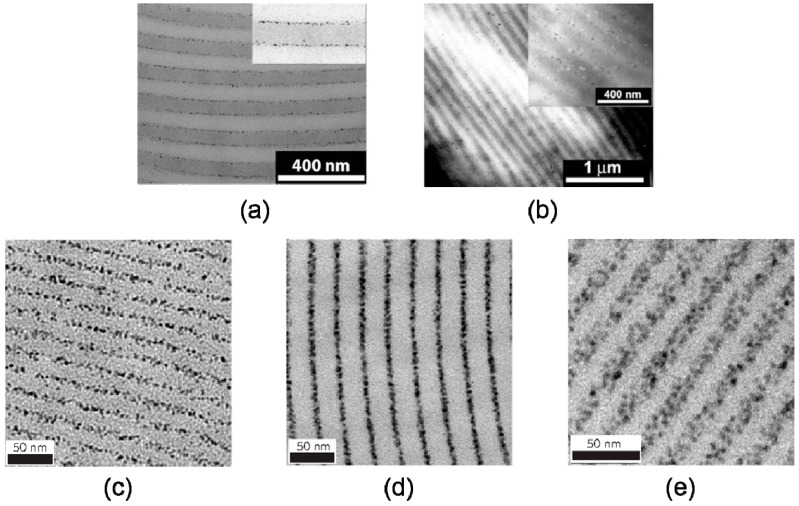
Experimental findings of size-dependent localization and dispersion of nanoparticles. Small PS-coated gold nanoparticles dispersed in a PS–PEP thin film, (**a**) localized near the interfacial regions, while large silica nanoparticles (**b**) concentrate in the middle region of PS domain. Reproduced with permission from ref. [55] Copyright 2003, The American Chemical Society. As the temperature rises, spatial distribution of nanoparticles dispersed in PS-P2VP (3-PDP) varies from (**c**) in the middle region (50 ℃), (**d**) at the interface (110 ℃) to (**e**) uniformly distributed (160 ℃). Reproduced with permission from ref [14] Copyright 2017, The Springer Nature.

Block copolymer nanocomposites is another typical system, where such nanoparticle size-induced hierarchical structures have been discovered and researched theoretically as well as experimentally. With the help of self-consistent field/density field theories (SCF/DFT), Thompson et al. [12] predicted that larger selective nanoparticles localize at the center of the preferred phase, whereas smaller particles are more uniformly dispersed within a specific phase, shown in Figure 4a. Lee et al. [68] decomposed the free energy into contribution of enthalpy, translational entropy, and conformational entropy using strong segregation theory. The results revealed that the enthalpic contribution to the free energy was more and more significant when small nanoparticles migrated into the enthalpically unfavorable regions. Bockstaller and coworkers [69] studied the localization of small PS-coated gold nanoparticles with a radius of 2.5 nm and large silica nanoparticles with a radius of 22nm in the microphase-separated polystyrene-b-poly(ethylene propylene) (PS-PEP) diblock copolymer matrices. The experimental observations by TEM obtained the same morphological characteristics as the theoretical results, shown in Figure 5a,b. 

Tailored by the enthalpy–entropy compensation, an approach to generate stimuli-responsive materials has been achieved, which is versatile and applicable to a wide range of nanoparticles and polymer matrices. Zhao et al. [13] reported that blends of diblock copolymers (PS-b-P4VP), small molecules (3-PDP), and nanoparticles (CdSe, PbS or Au) formed a lamellae-within-lamellae hierarchical structures in one-, two-, or three-dimensional arrays, relying on the volume fraction of 3-PDP and nanoparticles. Moreover, the spatial distribution of nanoparticles was a function of temperature, causing reversible thermoresponsive property, shown in Figure 5c-e.

Above all, examining size-dependent localization of nanoparticles in a block copolymer template can lead to the conclusion that small nanoparticles are located near the block copolymer domain interface while large nanoparticles are located in the center of their preferential block copolymer domain [53].

### 3.2. Volume Fraction

**Figure 6:**
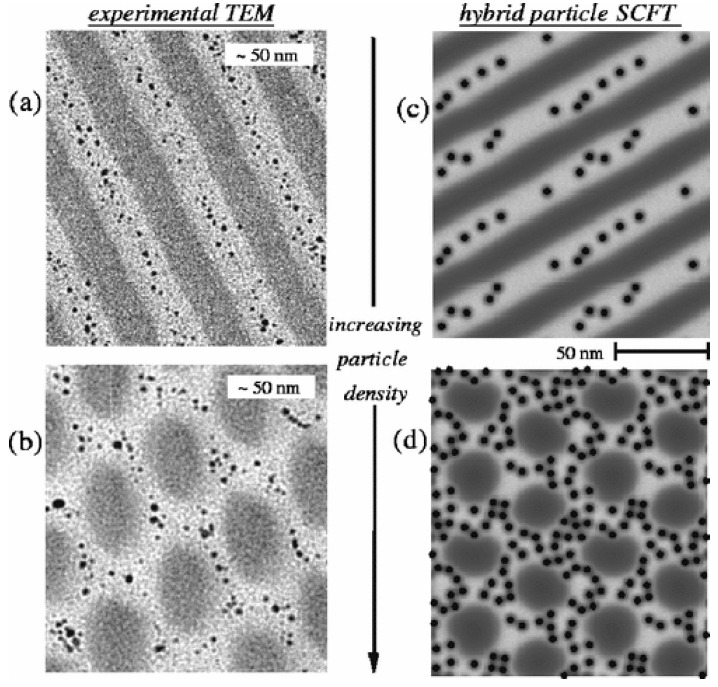
TEM images of *f*_PS_ = 0.50 PS-P2VP diblock nanocomposite containing PS-functionalized Au particles with particle volume fraction (**a**) 0.10 and (**b**) 0.35. Total particle size including the PS shell is 2.6 times that of the Au cores seen as black dots in the TEM images. Hybrid particle-field (HPF) simulation results (right column) show monomer volume fractions representing PS (light), P2VP (dark), particles (black). Simulation parameters: *f*_PS_ = 0.5, Flory parameter (*χ*) for PS-P2VP diblock *χ* = 0.16, *λ* = 0.16, and particle area fraction (**c**) 0.04 and (**d**) 0.18. The particle configurations shown are representative of those obtained based on several independent runs for a given nanoparticle density. Reprinted with permission from ref. [71] Copyright 2006 by the American Physical Society.

The volume fraction of nanoparticles dispersed in the polymer matrices can influence the polymer conformational entropy, particle translational entropy, and enthalpy, which may eventually lead to order-to-order (OOT) and order-to-disorder (ODT) phase transitions, generating various ordered and disordered morphologies [49]. As shown in Figure 3e, the large nanoparticles selectively distributed in the preferential domain of the block copolymer, self-assembled to gain excess entropy as the volume fraction increased. Kim et al. [70] reported a lamellar-to-cylindrical phase transition occurring in a polymer blend of PS-b-P2VP and PS-coated gold nanoparticles with a radius of 2.5 nm. The cross-sectional transmission electron microscopy (TEM) images in Figure 6a,b showed the morphologies of lamellar phase for *ϕ*_p_<*ϕ*_cri_ and cylinder phase for *ϕ*_p_>*ϕ*_cri_. For cylinder phase, small nanoparticles tended to be uniformly distributed in the PS domain, while large nanoparticles located at the center of PS domain), which is in agreement with the respective localization of nanoparticles in the lamellae phase. Sides et al. [71] performed a 2D simulation with the hybrid particle-field (HPF) algorithm, underestimating the particle concentration at the morphological transitions, as shown in Figure 6c,d. Qualitatively similar predictions have also been reported by Xu et al. [72] using self-consistent field theory, showing that those structural transitions are dictated by the competition between entropy and enthalpy.

### 3.3. Shape

The entropic effect of incorporation of anisotropic nanoparticles is more complicated than that of spherical nanoparticles. On one hand, rotational entropy has to be taken into consideration, as a result of the broken symmetry [22,33]. On the other hand, there exist several evidences that the emergence of directional entropic forces due to particle anisotropy has the potential to direct the phase morphologies [34,35,36].

**Figure 7:**
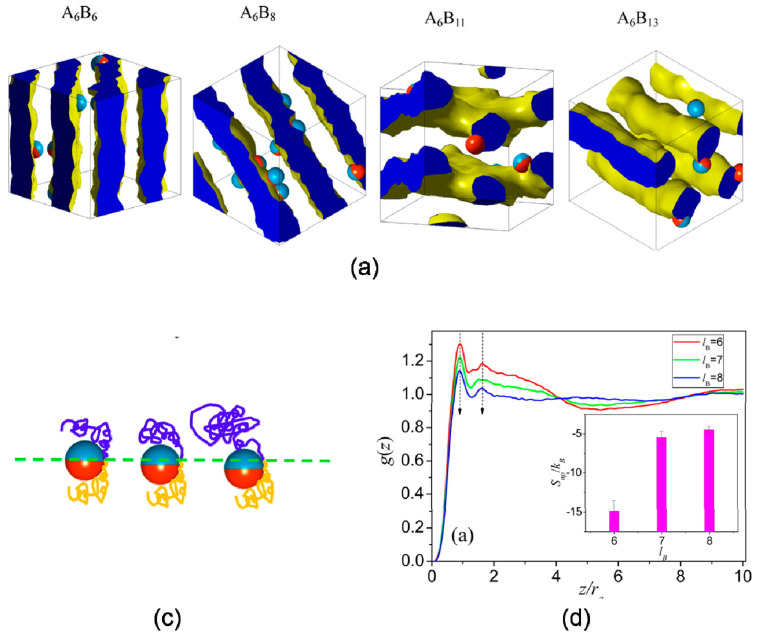
Janus particles assemble in asymmetric diblock copolymer. (a) Typical simulated morphologies formed by the self-assembly of Janus nanoparticles in various diblock copolymers. (b) Schematic snapshots of particle and surrounding chains at various *l*_B_, and (c) excess entropy per bead *S*_BB_ estimated from the pair correlation function. Reprinted with permission from ref. [11] Copyright 2014 by the American Chemical Society.

Dong et al. [11] studied entropy-mediated interfacial organization of Janus nanoparticles in symmetric and asymmetric diblock copolymer melts, revealing an unconventional entropy effect that explains the morphologies and interfacial behaviors of the nanoparticles. Increasing the length of B segments leads to topology mismatching between Janus nanoparticles and polymer interfaces at the mesoscale, as shown in Figure 7a. To prevent high free energy at unfavorable regions, B segments stretched around the particle before the phase transition, which accounted for position transition of Janus particle at interface, as shown in Figure 7b,c. 

**Figure 8:**
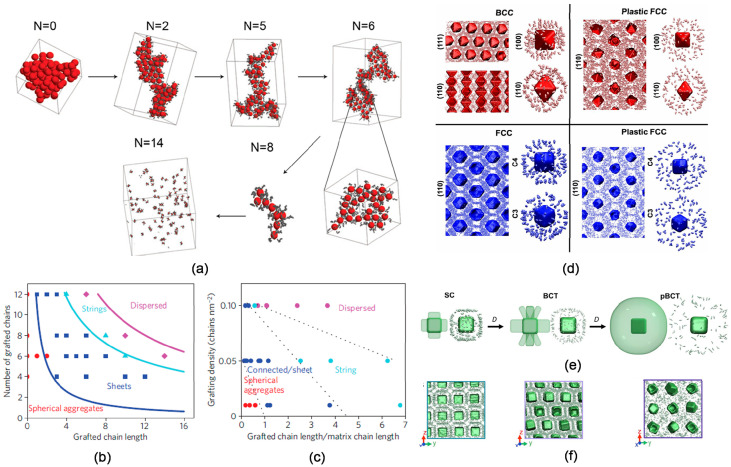
(a)–(c) Anisotropic structures of hieratical self-assembly of spherical polymer-grafted nanoparticles (**a**) Simulation snapshots of particles with different grafted chain length *N*, where six polymer chains were uniformly grafted onto spherical surface. Phase diagram of polymer-grafted particle obtained from (**b**) numerical simulations and (**c**) experiments, with grafted chain length versus number of grafted chains (grafted density), where the morphologies are classified by color symbol: spheres (red), sheets (dark blue), strings (light blue), and well-dispersed particles (magenta). The solid or dash lines that separate the different regions are merely guides to the eye. Reproduced with permission from ref. [73] Copyright 2015, The Springer Nature. (d)–(f) MD simulations of self-assembly of DNA-sticky polyhedral. (**d**) The spatial distribution of DNA sticky ends is shown for BCC, plastic BCC, FCC, and plastic FCC phase, where the DNA is localized between particles for the crystalline phase and more isotropically distributed for the plastic phase. (**e**) A model (left) and image (right) from an MD simulation are shown to indicate the distribution of DNA sticky ends in each plane, as *D* increases in these systems, where the distribution of DNA ligands breaks symmetry along the (001). (**f**) MD simulations along particular crystallographic planes reveal the distribution of DNA sticky ends between particles. Planes shown in (e) and (f) are (100) for SC, (100) for BCT, and (110) for the plastic BCT. Reproduced with permission from ref. [74] Copyright 2016 National Academy of Sciences.

Akcora et al. [73] studied amphiphile-like behaviors of polymer-grafted nanoparticles through considering a series of anisotropic structures of self-assembly in experiments and theoretical simulations. Upon increasing the length of grafted chains, a trend can be observed from first spherical segregation clusters to large sheet-like clusters, strings and finally isolated particle structures, as indicated in Figure 8. The results suggested the morphologies balance the interaction energy gain when particle cores approached and the entropy loss of distorting the grafted polymers. Those approaches to anisotropic nanoparticle self-assembly may offer an alternative for reinforcing mechanical properties. 

Self-assembly of polyhedrals suggests that directional interactions induced by shape entropy should produce a wide variety of complex structures [34,35,36,37]. For example, O’brien et al. [74] investigated the crystallization behavior of polyhedrals assembled via the directional interaction of DNA surface ligands, revealing the “anisotropy zone” originated from an unexpected symmetry breaking in the DNA ligand distribution. As the DNA length *D* increased, less electrostatic repulsion (enthalpic contribution) and greater free volume available to DNA ligands (entropic contribution) accounted for the phase transitions from crystal to plastic crystal (Figure 8d), rather than the packing of the particle shape. Those experimental findings and theoretical simulations establish the range of conditions where shapes of both the polymeric and particulate entities can be harnessed to fabricate nanostructured materials with hierarchical order [75,76,77].

## 4. Entropy-Induced Transition by External Conditions

Entropy plays an important role in the structural formation of nanocomposites, offering us a new avenue to regulate the mesoscopic structures. However, equilibrium is a delicate balance in nature, where most of applications of nanocomposites can’t reach in practice. Of particular interest and importance in the study of polymer nanocomposite systems is clarifying the formation of the mesoscopic structures and non-equilibrium states under various external conditions such as confinement, external field, heating/cooling, and reaction.

### 4.1. Confinement

Many systems of biophysical and technological interest consist of nanoparticles and polymer chains in a confined volume, where not only interactions between polymers and particles but also the volume limitations need to be taken into considerations [51,78,79,80]. One key study [68] relating confinement to diblock copolymer nanocomposite is that reported by Lee et al., where they prepared blends of AB diblocks and nanoparticles that are confined between two hard walls separated by a distence using SCFT/DFT calculations. In this study, a complex interplay of entropic and enthalpic interactions drives the nonselective particles to localize at the hard confined surfaces, causing the blends to spontaneously self-assemble into particle-decorated lamellae that are oriented perpendicular to the surfaces. They calculated the free energy as a function of surface separation distance and made a comparison between the filled system and an unfilled one, demonstrating that nanoparticles extended the region of stability of the perpendicular phase [81].

Taking inspiration from the confinement effect, a comprehensive series of theoretical simulations [82,83,84] were conducted to study diffusion behavior of nanoparticles in nanocomposite materials with cracks, predicting that the depletion attraction between particles and surface will drive nanoparticles into them. Though driving the particles to this interface, the loss of conformational entropy of polymer chains was minimized, because they didn’t have to extend around the particles [1]. Gupta et al. [41] proposed a bilayer containing a PMMA glass mixed with PEG-grafted CdSe nanoparticles coated on a silica oxide wafer with cracks. As the temperature increased, nanoparticles swelled in the polymer melt migrated to the cracks. Migrating speed was determined by temperature and the radius of gyration of polymer chains, relating to particle motion and entanglment of polymer chains.

### 4.2. External Field

**Figure 9:**
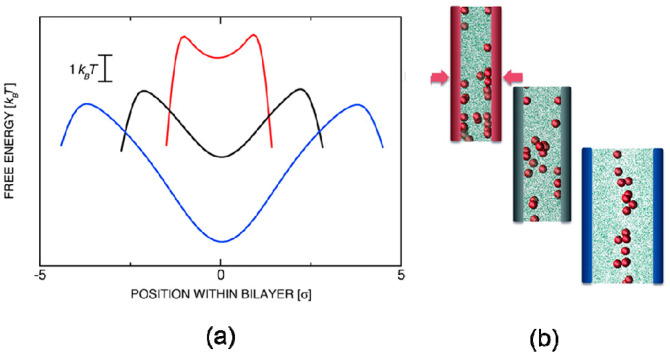
Nanoparticles confined between two polymer-grafted surfaces with disordered grafting obtained by Monte Carlo (MC) simulations, where (**a**) Free-energy as a function of position within bilayer (**b**) Schematic illustration of spatial distribution of nanoparticles. Reproduced with permission from ref. [85] Copyright 2014, The American Chemical Society.

In the mechanical process, blends of polymer and nanoparticle usually suffer from external pressure, whereas the deformation of polymer chains leads to change of conformational entropy. Curk et al. [85] employed MC simulations to evaluate the single-particle insertion free energy profiles between two polymer-grafted layers, showing that global and metastable minima of free energy were inverted by applied mechanical pressure in Figure 9. Few nanoparticles concentrated in the middle of the polymer-grafted sandwich with low external load applied. To overcome the external pressure, the grafted polymer chains had to deform and stretch around nanoparticles, associated with a great loss in conformation entropy, forcing nanoparticles to move from the center to the interfacial region.

Cui et al. [86] synthetized a surfactant based on the self-assembly of a hydrophilic nanoparticle with a functionalized oleophilic polymer at a water-oil interface. The number of polymer chains contacted with nanoparticle was controlled by the minimum in the free energy, a balance of the interfacial energy, and conformation entropy of polymer chains. When a drop was deformed by adding electric field, surface area increased as well as the free volume available to polymer chain, and thereby more surfactants could assemble at the surface to maintain non-equilibrium shape.

### 4.3. Heating/Cooling

Polymer nanocomposites can respond to temperature in some intriguing and counter-intuitive manners in both theoretical simulations and experiments. Taylor et al. [87] investigated the blend of PMMA colloid (diameter *d* = 1080 nm) and PS polymer (radius of gyration under *θ* conditions *R_g_* = 95 nm), which was observed to be ‘quenching’ by heating and ‘melting’ by cooling. When cooling the system, a fluid–gel transition occurred at room temperature, where volume fraction *ϕ*_C_ < *ϕ*_Cr_. The effective attraction between colloids induced by polymer depletion force was reduced, because the polymer radius of gyration decreased as the *θ*-temperature was approached, which raised the effective temperature, leading to ‘melting’ of colloidal gels. Similarly, Cao et al. [88] simulated the mixture of nanoparticles and polymer confined between sandwiched substrates, demonstrating that nanoparticles aggregated in the center at a low temperature, but were absorbed by substrate and created a ‘crystalline’ layer at a higher temperature. The inverse temperature crystallization of nanoparticles was also originated from the entropic depletion attraction between nanoparticle and the substrate, which leads to a gain of free energy.

**Figure 10:**
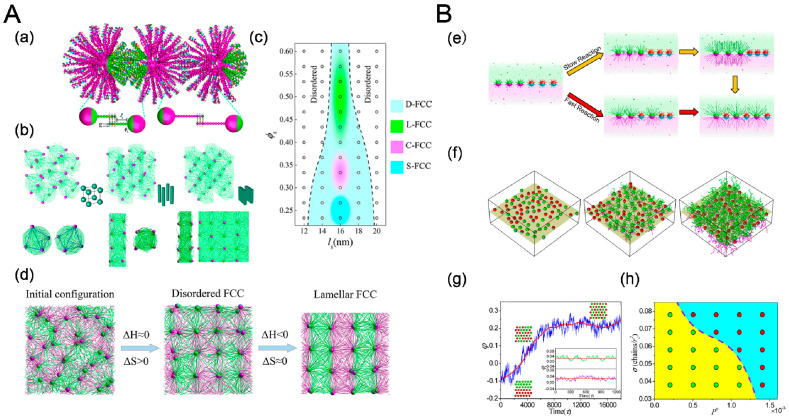
(**A**) Self-assembly of Janus particles (JNPs) with DNA hybridization and its crystalline process. (a) Schematic illustration of coarse-grained DNA-mediate Janus particles and their hybridization. (b) Hierarchical crystalline structures formed by self-assembly of DNA-JNP: Simple cubic (left), tetragonally ordered cylinder (middle) and lamellae(right), and (c) the corresponding phase diagram. (d) Representative structures during the formation of L-FCC at different stages: (left) disordered structure at the initial stage; (middle) D-FCC crystal at an interim stage and (right) L-FCC crystal at the late period. Reproduced with permission from ref. [90] Copyright 2018, The American Chemical Society. (**B**) Polymerization of grafted Janus nanoparticles at interface. (e) Schematic representations of the transition from randomly mixed phase to intercalated phase in the mixtures of grafted Janus nanoparticles, where one component undergoes, respectively, slow and fast polymerization reactions initiated from nanoparticle surfaces. (f) Temporal evolution of the mixing parameter, *φ*, during the reaction. (g) Typical interfacial nanoparticle organizations obtained at different stages of the reaction. (left) initially random configuration at 0τ, (middle) medium stage at 4000τ, and (right) late stage at 10,000τ. (h) State diagram of phase transition as a function of the propagation probability and the grafting density. The green and red circles indicate the first-order and second-order phase transitions, respectively. Reproduced with permission from ref. [91] Copyright 2018, The American Chemical Society.

Crystallization behavior of sequence-specific DNA hybridization of Janus particles (JNPs) has been studied recently by MD simulations [89], revealing tetrahedral nanoclusters and diverse secondary crystalline phases including simple cubic (SC), tetragonally ordered cylinder (P4), and lamella (L) structures, as shown in Figure 10a. The structures of crystalline depended on length of DNA sequence *l*_s_ and surface fraction of A-DNA chains *ϕ*_s_ associated with DNA-JNP’s anisotropy (Figure 10b). For small *l*_s_, tetrahedral nanoclusters occupied high symmetry areas, accounted for conformation entropy penalty originated from bounded DNA chains [90]. For large *l*_s_, the cooling crystallization dynamics of the hierarchical DNA-JNP crystals included two consequent processes: entropy-dominated translational order for the primary crystalline structure and enthalpy-dominated rotational order for diverse secondary crystalline structures. 

### 4.4. Reaction 

Chain-growth polymerization processes are usually accompanied by transformation of morphologies, which relates to translational entropy and conformation entropy. Yang et al. [91] studied the Janus particles at a fluid–fluid interface with polymerization reaction initiated from the surfaces of just one component of nanoparticles. At low reaction rates, a sharp increase in the mixing parameter *ϕ* was observed during the process, which was similar to first-order, equilibrium phase transition, as shown in Figure 10e,g. By increasing the reaction rate, the increase in the mixing parameter become smoother, behaving as a second-order-like phase transition. This reaction- induced phase transition from randomly mixed phase to intercalated phase was attributed to a gain in conformational entropy of growing chains overwhelming the direct contact chains repulsive energy in the initial stage, which however was inverted at the later stage. The ‘cage’ effect, where diffusion of nanoparticles was restricted by growing chains, significantly impacts the dynamics of phase transition. Only in fast reaction condition the nanoparticle could get remarkable energy to jump out of the ‘cage’ continually. 

Recently, some groups are incorporating entropy considerations and size/shape effects to combine various nanoparticles into hybrid systems. In this way, they have been able to experimentally and theoretically improve different properties of polymer nanocomposites. For example, the importance of the relationship between dispersion and orientation of graphene nanoribbon and electrical properties has been highlighted by the research group of Sundarara [17,18], and was supported by DPD simulations [75,76,77]. Confinement effects in highly-filled polymer composites and nanocomposites are also an important field where energy/entropy considerations play a key role in determining the final structures and properties [78,79,80]. For instance, the interactions and conformational characteristics of confined molten polypropylene (PP) chains between ferric oxide (Fe_2_O_3_) substrates were investigated by molecular dynamics (MD) simulations, showing the influence of the confinement on the buildup imbalance of normal and tangential pressures [78]. 

## 5. Conclusions

This review has summarized past and recent researches of various aspects of entropic effects in polymer nanocomposites. Our objective is to provide a qualitative and even quantitative understanding of the entropic contribution in the nanostructure transition processes and to help development of design strategies and guidelines. Simulation and theory promise to be a valuable tool not only for the investigation of thermodynamics and kinetic processes over a considerable range of time and length scales, but also for the preparation of epitaxial nanostructures as aforementioned description. The examples mentioned above also emphasize that delicate nanostructure can be precisely tailored by a proper size, shape and concentration due to various forms of entropy, with a good agreement between experimental findings and insight obtained from simulations and theoretical calculations [51,53,69]. This provides a fascinating approach to design and synthesize such materials with superior physical properties and multiple functions. However, by slightly changing the synthesis parameters, the same nanocomposite systems can yield different structures when pushed away from equilibrium [69]. In this regard, thermodynamics and kinetics properties of polymer nanocomposites in non-equilibrium processes, such as freezing process, sol–gel process and so on, remains to be further explored to extend our knowledge of fascinating and designed nanostructures
